# Outcomes in the Utilization of Single Percutaneous Cholecystostomy in a Low-Income Population

**DOI:** 10.3390/ijerph14121601

**Published:** 2017-12-19

**Authors:** Ping Lu, Nan-Ping Yang, Nien-Tzu Chang, K. Robert Lai, Kai-Biao Lin, Chien-Lung Chan

**Affiliations:** 1 School of Economics and Management, Xiamen University of Technology, Xiamen 361024, China; luping@xmut.edu.cn; 2 Department of Information Management, Yuan Ze University, Taoyuan 32003, Taiwan; 3 Department of Surgery, Keelung Hospital, Ministry of Health and Welfare, Keelung 20147, Taiwan; yang.nanping@gmail.com; 4 Institute of Public Health, National Yang-Ming University, Taipei 11221, Taiwan; 5 School of Nursing, College of Medicine, National Taiwan University, Taipei 10051, Taiwan; ntchang@ntu.edu.tw; 6 Department of Computer Science and Engineering, Yuan Ze University, Taoyuan 32003, Taiwan; krlai@cs.yzu.edu.tw (K.R.L.); kblin@xmut.edu.cn (K.-B.L.); 7 Innovation Center for Big Data and Digital Convergence, Yuan Ze University, Taoyuan 32003, Taiwan; 8 School of Computer and Information Engineering, Xiamen University of Technology, Xiamen 361024, China

**Keywords:** health insurance, percutaneous cholecystostomy, acute cholecystitis, low-income population, socioeconomic status

## Abstract

Numerous studies have investigated the applicable populations for percutaneous cholecystostomy (PC) procedures, but the outcomes of PC in low-income populations (LIPs) have been insufficiently studied. Data for 11,184 patients who underwent PC were collected from the National Health Insurance Research Database of Taiwan during 2003 and 2012. The overall crude rate of single PC for the LIP was 64% higher than that for the general population (GP). After propensity score matching for the LIP and GP at a ratio of 1:5, the outcome analysis of patients who underwent PC showed that in-hospital mortality was significantly higher in the LIP group than in the GP group, but one-year recurrence was lower. The rates of 30-day mortality and in-hospital complications were higher for the LIP patients than for the GP patients, and the rate of routine discharge was lower, but the differences were not significant. In conclusion, LIP patients undergoing PC exhibit poor prognoses relative to GP patients, indicating that a low socioeconomic status has an adverse impact on the outcome of PC. We suggest that surgeons fully consider the patient’s financial situation during the operation and further consider the possible poor post-surgical outcomes for LIP patients.

## 1. Introduction

The mainstay of therapy for acute cholecystitis (AC) is cholecystectomy [1]. However, the morbidity and mortality rates are high in elderly patients and in those with comorbidities at the time of surgery [2,3]. An alternative, safe treatment for acute inflammation of the gall bladder is percutaneous cholecystostomy (PC) procedures, which involves percutaneous, imaging-guided catheter placement in the gallbladder lumen; PC was first described by Radder in 1980 [4]. PC is a less invasive method than cholecystectomy for treating AC in patients who are critically ill or have serious medical comorbidities precluding the use of general anesthesia [5]. This procedure allows for the immediate decompression of an acutely inflamed gallbladder and requires the use of only local anesthesia, thus eliminating the need for surgery; moreover, the procedure can serve as either a bridge to surgery or a definitive treatment designed for unfit patients and for those who refuse to undergo cholecystectomy [6].

Numerous studies have investigated the applicable populations for PC procedure, and most of these studies have concluded that PC is a valuable and effective procedure for the population of critically ill and elderly patients [7,8]. In addition, several studies have also addressed the outcomes of PC in specific populations, such as burn patients [9,10], veteran patients [11], and pregnant patients [12,13]. However, to our knowledge, the outcomes of PC in low-income populations (LIPs) have been insufficiently investigated. Additionally, many studies have examined the factors that influence the effectiveness of PC treatment, but few studies have addressed the effects of socioeconomic status (SES) on the PC outcome. Numerous studies have shown that in several western countries, SES has the strongest association with postoperative mortality [14,15,16]. Several previous studies have also shown that a low SES is linked to impaired access to surgical care and that delayed treatment is a strong risk factor for the poor outcomes of operations, such as cholecystectomy, associated with gallstone pathogenesis [17,18]. Furthermore, after conducting six surveys regarding the living conditions of the LIP and moderately LIP, the Taiwan Ministry of the Interior has concluded that the LIP is more susceptible to serious disease than the general population (GP) in Taiwan [19,20,21]. Therefore, an examination of the outcomes of PC in an LIP, per a population-based dataset, is necessary, and the results may lead to treatment suggestions for medical research institutions and assist surgeons in making decisions.

## 2. Materials and Methods

### 2.1. Study Subjects and Data Source

All data analyzed in this research were obtained from the National Health Insurance Research Database (NHIRD). The academic databank of NHIRD included various sub datasets, such as the inpatient expenditures by admission (DD), details of prescription orders (DO), and ambulatory care expenditures by visit (CD). In this study, the DD dataset was used for further analysis.

### 2.2. Data Protection and Permission

This study was approved by the research ethics committee of Taoyuan General Hospital (Approval Number: TYGH103015), which has been certified by the Ministry of Health & Welfare of Taiwan, and the research protocol was reviewed by the National Health Research Institutes (Agreement Number: NHIRD-104-081). For protection of personal information, all patient information was double encrypted to protect the patient privacy. Moreover, any researcher who used the NHIRD was required to declare and sign a written agreement.

### 2.3. Data Definition

In this study, we focused on the outcomes of the utilization of PC in an LIP. The International Classification of Diseases, 9th Revision, Clinical Modification (ICD-9-CM) diagnosis codes and ICD-9-CM treatment codes were evaluated. PC was identified as ICD-9-CM procedure code 51.01 [22]. The reasons for the procedure were analyzed as follows: ACs with a calculus/stone were defined as patients with ICD-9-CM diagnosis codes 574.0, 574.3, and 574.6; ACs without a calculus/stone were defined as patients with ICD-9-CM diagnosis code 575.0; calculus without ACs defined as patients with ICD-9-CM diagnosis codes 574.1, 574.2, 574.4, 574.5, 574.7, 574.8, or 574.9; other disorders of the gallbladder or biliary tract were defined as patients with ICD-9-CM diagnosis codes 575 or 576, excluding diagnosis code 575.0; and malignant neoplasms of the digestive organs and the peritoneum included patients with ICD-9-CM diagnosis codes 150–159 but omitted diagnosis codes 574, 575, and 576.

### 2.4. Patient Selection

We used the following inclusion and exclusion criteria: (1) Patients who initially underwent PC from 1 January 2003 through 31 December 2012 were retrieved from the NHIRD; (2) Patients aged less than 18 years old were excluded; (3) Patients who underwent both PC and cholecystectomy operations during the same hospitalization were excluded.

**Figure 1:**
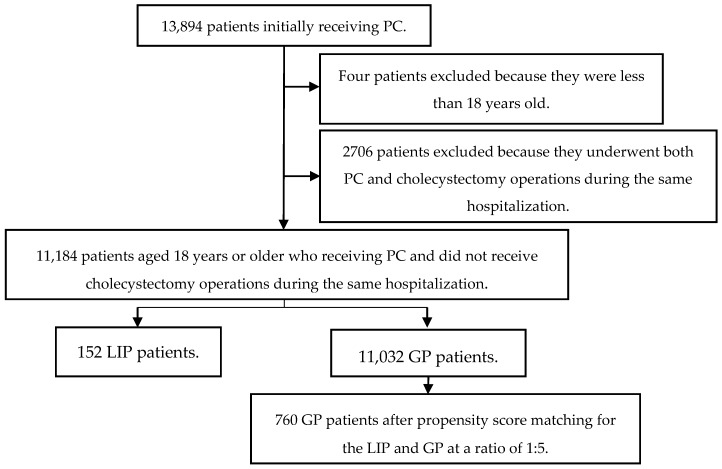
Patient selection flowchart. PC: percutaneous cholecystostomy; LIP: low-income population; GP: general population.

To evaluate the effects of SES, the enrolled subjects were divided into GP and LIP groups based on whether they satisfied the criteria of Taiwan’s Public Assistance Act [23]. The LIP was recorded as the fifth class insured in the NHI database [24]. The GP comprises individuals who were not classified in the LIP. To reduce selection bias, we adopted propensity score matching (PSM) to select the control group at a ratio of 1:5. Figure 1 shows the flowchart of patient selection.

### 2.5. Measurement Outcomes

#### 2.5.1. Thirty-Day Mortality and In-Hospital Mortality

Thirty-day mortality referred to patients who died within 30 days after discharge. In-hospital mortality referred to patients undergoing PC who died during hospitalization.

#### 2.5.2. In-Hospital Complications

We examined all-cause, nonfatal in-hospital morbidity rates based on the ICD-9-CM codes. Complications were grouped into nine categories: mechanical wound complications, infections, urinary complications, pulmonary complications, systemic complications, complications arising during the procedures, specific complications of the gallbladder/digestive system, respiratory complications, and other. Because the NHIRD DD dataset included inpatient data only, complications occurring after hospital discharge were not considered in our analysis.

#### 2.5.3. Routine Discharge

The NHIRD provided information on patient discharge statuses as follows: 1, treated and discharged; 2, continued in-hospital treatment; 3, transferred to outpatient treatment; 4, death; 5, discharge against medical advice; 6, referral; 7, change in status; 8, absconded; 9, suicide; 0, other; and A, discharged while dying (the patients leaving against medical advice were usually in critical status and always received home hospice care in a Chinese-cultural society). Patient categories were grouped as follows: routine discharge (1, 3) and non-routine discharge (0, 2, 4–9, and A).

#### 2.5.4. One-Year Recurrence

One-year recurrence was designated when readmission occurred due to the diagnosis of cholecystitis after PC within one year.

### 2.6. Statistical Analysis

For the analysis, descriptive statistics for comparing baseline characteristics were determined based on the number of cases, percentages, and 95% confidence intervals (CIs) for the estimated rates. Chi-square tests and independent *t*-tests were used to evaluate the significance of the frequency differences and continuous variable differences, respectively, between two subgroups. PSM was used in the observational studies to reduce selection bias by matching the different groups based on the propensity score probabilities [25]. The binary logistic regression analysis method was used, and the adjusted odds ratio (OR) was calculated. Temporal trends were analyzed using joinpoint regression, a statistical method that fit a series of joined straight lines between statistically significant changes in trends (joinpoints). In turn, we estimated the change between the joinpoints using the National Cancer Institute’s Joinpoint Regression Program Version 4.3.1.0 [26,27]. Long-term trends over the entire time series were designated average annual percentage changes (AAPCs) and were estimated as the weighted average of short-term annual percentage changes (APCs) with weights equal to the length of the short-term line segment [28]. All statistical analyses were performed using Statistical Package for Social Sciences for Windows (SPSS for Windows Version 22.0, IBM Corporation, New York City, NY, USA).

## 3. Results

A total of 11,184 patients who underwent PC between 2003 and 2012 were retrieved from the NHIRD; the crude rate of single PC was 6.09 per 100,000 per year (95% CI: 4.56–7.62). Among them, 152 cases belonged to the LIP, and 11,032 belonged to the GP. The overall crude rate of PC for the LIP was 64% higher than that for the GP (LIP: 9.87 CI: 7.92–11.82 vs. GP: 6.01 CI: 4.49–7.53, per 100,000 per year). The mean age of the patients who underwent PC was 64.1 ± 16.0 in the LIP, which was significantly younger than the mean age of the GP (70.9 ± 14.6).

As shown in Table 1, the differences in sex, age stratum, and hospital level were significant between the LIP and GP (*p* < 0.005). Male patients accounted for 70.39% of all patients in the LIP who underwent PC, overwhelming the proportion of male patients in the GP (58.98%). Compared to the GP group, the LIP group included a high proportion of young patients (aged 30–39, 40–49, and 50–59) and a low proportion of old patients (aged more than 60). Particularly, the proportion of patients aged 60–69 and 70 years or more dominated the GP group at 61.79% and 16.82%, respectively. Therefore, the patients in the LIP who underwent PC were notably younger than the patients in the GP. Regarding the utilization of medical facilities, most of the GP patients (56.52%) underwent PC procedures in a medical center, while the majority of LIP patients (50.00%) underwent operations in a regional hospital; moreover, the proportion of PC procedures performed in district hospitals for the LIP patients was nearly 4-fold higher than the proportion for the GP patients (LIP vs. GP: 10.53% vs. 2.5%).

**Figure 2:**
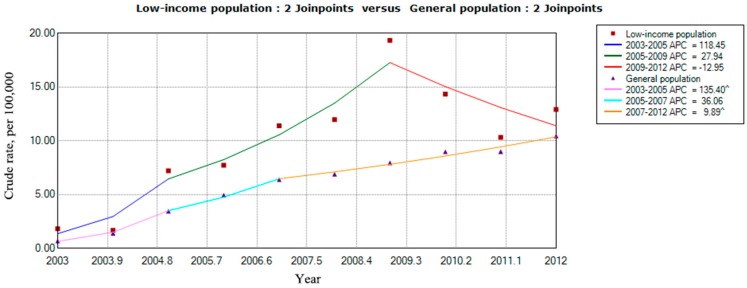
Comparison of the crude rates for the LIP and GP patients who underwent percutaneous cholecystostomy in Taiwan from 2003 to 2012.

During the observation period, the annual crude rate of LIP patients who underwent PC increased by 26.7% per year (AAPCs = 26.7%) from 1.91 to 12.92 per 100,000 per year, indicating a nearly 7-fold increase from 2003 to 2012. During the same period, the annual crude rate of GP patients who underwent PC increased by 36.5% (AAPCs = 36.5%) from 0.68 to 10.45 per 100,000 per year, indicating an increase of more than 15-fold from 2003 to 2012. The overall increasing trend for the crude rate of PC was slower in the LIP group than in the GP group, but the crude rate was obviously higher for the LIP group than for the GP annually. For the short-term trend, 2009 was an important turning point for the annual crude rate of patients in the LIP who underwent PC, and the crude rate increased up to 2009 but declined thereafter (APCs = −12.95%). In addition, the crude rate of patients who underwent PC in the GP maintained an increasing trend during the observation period (Figure 2).

To evaluate the outcome of PC in the LIP and avoid confounding bias, we adopted PSM to select the control group from patients in the GP who underwent PC. The logistics matching items were sex, age stratum, CCI score, cause of the procedures, and hospital level. The matching method was set as the nearest match, and the matching ratio was a 1:5 case-control match on the propensity score. After matching, no significant difference was observed regarding the demographic characteristics between the case and control groups, as shown in Table 2.

The main outcomes of PC are shown in Table 3, which shows the ORs and chi-square significance levels between the case and control groups. The logistics regression model was used to calculate the adjusted ORs and their significance levels. The in-hospital mortality rate for LIP patients was significantly higher than GP patients (adjusted OR = 1.816, *p* = 0.025). However, the one-year recurrence rate for LIP patients was lower than GP patients (adjusted OR = 0.583, *p* = 0.029). Of the 19 LIP patients who suffered from one-year recurrence of cholecystitis, 16 patients (84.21%) were receiving cholecystectomy, one patient (5.26%) was necessitating a further PC, the rest were treated by other conservative management; of the 176 GP patients, 132 patients (75%) were receiving cholecystectomy, 18 patients (10.23%) were necessitating a further PC, and the rest were treated by other conservative management. The outcome differences for 30-day mortality, in-hospital complications, and the rate of routine discharge were not significant. When utilization of hospital resources was assessed, no significant difference was discovered regarding the length of hospital stay (LOS) or hospital costs.

## 4. Discussion

Poverty is an ancient but persistent societal problem globally, and the understanding and management of poverty follows political, economic, social, demographic, and cultural transitions [29]. A clear definition of poverty is not easy because different societies take their own firm stances on the concept of poverty. In 1980, the government of Taiwan passed a law known as the “Public Assistance Act”, which has been amended several times according to social developments, to take care of the LIP and victims of emergencies or disasters and to enable independence [23]. Based on the latest version of the “Public Assistance Act”, low-income households were defined as those with an average per-person gross monthly income that was less than the monthly minimum living expense standard of the residential region. The minimum living expense standard was defined as 60% of the average monthly disposable income for each region. Family property was not permitted to exceed a certain amount, as determined by the central or municipal authorities in the corresponding year [23]. To ensure that the process and results were fair and equitable, the government would authorize special departments and staff to identify low-income households annually in Taiwan. After identifying the low-income households, the government not only offered economic and living benefits but also provided Medicaid. All subjects of this study were identified by the government of Taiwan based on strict standards and procedures, which enabled objective and rational identification of poor people. Thus, the quality of the current LIP data is reliable.

In our analysis, we found that the mean age of the patients in the LIP who underwent PC was 64.1 ± 16.0, which was significantly younger than the mean age of the GP (70.9 ± 14.6). Meanwhile, we also found that the crude rate of patients who underwent PC was significantly higher in the LIP than in the GP (LIP: 9.87 vs. GP: 6.01 per 100,000 per year, *p* < 0.001). Generally, PC is used for critically ill and elderly patients [30]. Thus, the young age and high crude rate of PC in the LIP indicate that when patients suffer from gallbladder disease, the proportion of critically ill patients becomes higher in the LIP than in the GP and that critically ill patients become more prevalent in the young LIP age groups, namely, 40–49 y/o and 50–59 y/o, than in the young GP age groups. Therefore, in the treatment of gallbladder disease, when encountering LIP patients, clinicians should exercise caution because LIP patients may be more vulnerable than GP patients, even among young patients. In addition, we found that the proportion of male patients who underwent PC was higher than that of female patients in the GP. Furthermore, the proportion of male patients who underwent PC in the LIP was much higher than that of female patients in the LIP, reaching more than two-fold that of the GP (70.39% for male vs. 29.61% for female); the reason behind this observation needs further study. The proportion of LIP patients who underwent PC in a medical center was significantly lower than the proportion of GP patients (39.47% for LIP vs. 56.25% for GP, *p* < 0.001). Furthermore, the LIP patients were more inclined than the GP patients to utilize a regional or district hospital, accounting for 60.53% of the total LIP (Table 1). This result indicated that the LIP patients were at a disadvantage regarding access to medical resources compared to GP patients. The reason may be that some LIP patients live in more remote areas than GP patients do, and the location may make travel to a medical center inconvenient.

The crude rate of patients who underwent PC in the GP showed a steady upward trend (Figure 2), which was consistent with several previous investigations [7,31,32]. Richard et al. [31] reported that the annual PC procedures performed on Medicare beneficiaries increased by almost 6-fold between 1994 and 2009 in the United States. Travis et al. [32] reported an increased use of PC for the treatment of AC over a 20-year period at a single institution. The marked increase in the utilization of PC over time may be caused by three reasons. First, the aging of the population in Taiwan may have affected the increase in PC use, as PC has been established as a treatment option for AC among elderly and critically ill patients [32,33], and the number of elderly and critically ill patients may increase as the elderly population ages. Second, clinical acceptance and the availability and expertise of interventional radiologists may also contribute to the growth of PC use [31]. Finally, the Tokyo guidelines, first in 2007 and then again in 2013, considered the use of PC not only as an alternative procedure in critically ill patients but also as a bridge to surgery in patients with moderate-grade cholecystitis [34,35]; this may also have affected the growth trend of PC use. The crude rate of LIP patients who underwent PC also increased from 2003 to 2009, reaching its peak in 2009. However, the crude rate showed an obvious downward trend from 2010 to 2012 (APCs = −12.95). The reason may be the revision of the “Public Assistance Act” in 2010. To expand the care of vulnerable people and to enhance the effectiveness of the new social assistance system, this version of the “Public Assistance Act” adjusted the poverty line, lowered the threshold for inclusion, increased the social assistance provisions of the notification mechanism, and increased cooperation with non-governmental organizations [21]. These changes caused the number of low-income households to reach 144,186 by the end of June 2013, constituting an increase of 56,521 or 64.5% from the end of June 2008 [21]. These measures increased the overall population of low-income people to a certain extent and narrowed the crude rate of PC among the LIP and GP.

We observed that the in-hospital and 30-day mortality rates were 11.93% and 4.41%, respectively, for all patients who underwent PC. Numerous studies have concluded that PC is a safe procedure with a low overall mortality rate [36,37], so the in-hospital and 30-day mortality rates of the LIP and GP patients were high in the present study; however, similar mortality rates have emerged in several other studies [32,38,39,40]. For example, Anders et al. [40] reported that the 30-day mortality rate was 15.4% in patients treated with PC. Travis et al. [32] reported a 30-day mortality rate of 36% from March 1989 to March 1998 and of 12% from April 1999 to April 2009. Because the majority of patients who underwent PC were elderly or critically ill, further study of whether the deaths were caused by the PC itself was necessary. In our analysis, we found that a large proportion of patients who underwent PC had a primary diagnosis code that was not gallbladder disease (46.88% of the LIP patients and 52.26% of the GP patients), and a large portion of these patients had primary procedure codes that were not PC (34.38% of the LIP patients and 47.74% of the GP patients). In the NHIRD inpatient database, the diagnostic and surgical code for each record was five, indicating that the cause of death for a large number of the patients who died may not have been the PC operation itself and that the deaths may have been caused by other diseases or surgeries. This situation has been reported in several studies [36,39,41]. For example, Li et al. [36] reported that most deaths in their study were due to unrelated illnesses. Nasim et al. [39] summarized that the 30-day mortality rate of PC for AC ranged from 18% to 69%, which was attributed to the presence of co-morbid conditions in the selected patients.

The reason for the high number of deaths for patients who undergo PC needs further study. However, we were more concerned about whether the in-hospital and 30-day mortality rates for the LIP patients differed from those of the GP patients under the same conditions. As shown in Table 1, significant differences between the LIP and GP patient groups were evident for several demographic characteristics, such as sex, age, and hospital level, so the comparisons for the in-hospital and 30-day mortality rates between the LIP and GP groups were inaccurate. Therefore, to reduce selection bias, by matching subjects or patients on the probability that they would be assigned to a specific group, PSM was used to match LIP patients with GP patients, to ensure the homogeneity of the two groups. On this basis, we observed that the in-hospital mortality and 30-day mortality rates were significantly higher in the LIP group than in the GP control group, confirming that the LIP patients had more adverse treatment outcomes after PC than the GP patients under the same conditions. We also found that the number of in-hospital complications was higher for the LIP patients than for the GP patients and that the rate of routine discharge was lower for the LIP patients than for the GP patients; however, the differences were not significant.

As shown in Table 3, the one-year recurrence rate was 21.82% for patients who underwent PC in this study, which was slightly higher than the rates reported in some previous studies that ranged from 4% to 22% [38,42,43,44]. In addition, the one-year recurrence rate of patients who underwent PC for LIP patients was lower than that for GP patients (adjusted OR = 0.583, *p* = 0.029). The fact that the overall mean age of LIP patients was lower than that of GP patients may be one of the reasons for the lower one-year recurrence, although it could also be caused by other reasons; these warrant further research and in-depth clinical trials for verification, which we plan to conduct in the next phase of work. The LOSs of the patients who underwent PC were 18.26 ± 1.16 for the LIP and 16.37 ± 0.58 for the GP, which was consistent with previous reports indicating that the duration of drainage ranged from three to six weeks [43,44].

As with similar analyses using administrative and database-based studies, the present study has several inherent limitations. First, detailed clinical data and examination information could not be obtained in this study. As we could not review the individual medical records to ensure that the records were coded precisely, deviations may exist between the codes and the actual severity of the disease conditions. Second, our data contained only inpatient data, which was not connected to the government death registration data. Thus, the actual mortality rate may have been underestimated when the long-term mortality rate was calculated because we could not track the patients who died after discharge and who were not readmitted to the hospital before death. However, in reality, this situation happens rarely. We examined only the 30-day mortality rate in this study, and if the patient had a critical condition during the one month after the operation, he would have been sent to the hospital for re-examination according to the customs of Taiwan. Therefore, we would not have ordinarily missed this postoperative patient situation. Third, data on postoperative conditions were also not included. Even so, this national population-based claims database is recognized as reliable because the database has been adopted in many research fields and numerous high-impact publications. Meanwhile, the quality of the medical services provided to patients and the coding of those services were controlled through a uniform system, the National Health Research Database, which was built by the NHI Bureau. Therefore, the quality of the data used in this study is reliable.

## 5. Conclusions

In conclusion, the LIP patients who underwent PC procedures were younger, more likely to be male, and more likely to have surgery in a regional or district hospital than the GP patients. The LIP patients were associated with a significantly higher post-PC 30-day mortality and in-hospital mortality rates, but a lower rate of one-year recurrence. Generally, low SES has an adverse impact on the outcome of PC. We suggest that surgeons fully consider the patient’s financial condition during the operation and pay more attention to the possible poor post-surgical outcomes for LIP patients.

## Figures and Tables

**Table 1:** Demographic characteristics of patients undergoing percutaneous cholecystostomy in Taiwan from 2003 to 2012.

Variables	Low-Income Population (N = 152)		General Population (N = 11,032)		*p* Value
	*n*	%	*n*	%	
**Sex**					0.005
Female	45	29.61%	4525	41.02%	
Male	107	70.39%	6507	58.98%	
**Age Stratum**					<0.001
18–29 y/o	0	0.00%	111	1.01%	
30–39 y/o	8	5.26%	302	2.74%	
40–49 y/o	27	17.76%	614	5.57%	
50–59 y/o	31	20.39%	1336	12.11%	
60–69 y/o	19	12.50%	1856	16.82%	
70 y/o or more	67	44.08%	6813	61.76%	
**CCI Score**					0.445
0	72	47.37%	5422	49.15%	
1	46	30.26%	2782	25.22%	
2	19	12.50%	1407	12.75%	
≥3	15	9.87%	1421	12.88%	
**Cause of Procedure**					0.386
AC with a C/S	53	34.87%	4063	36.83%	
AC without a C/S	55	36.18%	3369	30.54%	
Calculus without AC	16	10.53%	1228	11.13%	
ODGBT	12	7.89%	1369	12.41%	
MNDOP	7	4.61%	347	3.15%	
Other	9	5.92%	656	5.95%	
**Hospital Level**					<0.001
Medical Center	60	39.47%	6205	56.25%	
Regional Hospital	76	50.00%	4551	41.25%	
District Hospital	16	10.53%	276	2.50%	

y/o: years old; AC with a C/S: Acute cholecystitis with a calculus/stone, AC without a C/S: Acute cholecystitis without a calculus/stone, Calculus without AC: Calculus without acute cholecystitis, ODGBT: Other disorders of the gallbladder or biliary tract, MNDOP: Malignant neoplasm of digestive organs and peritoneum, CCI: Charlson comorbidity index.

**Table 2:** Demographic characteristics of the patients undergoing PC after propensity score matching.

Variables	Low-Income Population (N = 152)		Control Group (N = 760)		*p* Value
	*n*	%	*n*	%	
**Sex**					1.00
Female	45	29.61%	225	29.61%	
Male	107	70.39%	535	70.39%	
**Age Stratum**					0.927
18–29 y/o	0	0.00%	4	0.53%	
30–39 y/o	8	5.26%	32	4.21%	
40–49 y/o	27	17.76%	126	16.58%	
50–59 y/o	31	20.39%	166	21.84%	
60–69 y/o	19	12.50%	99	13.03%	
70 y/o or more	67	44.08%	333	43.82%	
**CCI Score**					0.95
0	72	47.37%	377	49.61%	
1	46	30.26%	214	28.16%	
2	19	12.50%	97	12.76%	
≥3	15	9.87%	72	9.47%	
**Cause of Procedure**					1.00
AC with a C/S	53	34.87%	265	34.87%	
AC without a C/S	55	36.18%	278	36.58%	
Calculus without AC	16	10.53%	84	11.05%	
ODGBT	12	7.89%	56	7.37%	
MNDOP	7	4.61%	36	4.74%	
Other	9	5.92%	41	5.39%	
**Hospital Level**					0.865
Medical Center	60	39.47%	290	38.16%	
Regional Hospital	76	50.00%	397	52.24%	
District Hospital	16	10.53%	73	9.61%	

**Table 3:** Prevalence and odds ratios (ORs) for in-hospital mortality, 30-day mortality, in-hospital complications, routine discharge rate, and one-year recurrence among sampled subjects after propensity score matching.

Outcome Variable	Total (N = 912)		Low-Income Population (N = 152)		Control (N = 760)		*p* Value ^a^
	*n*	Percent (%)	*n*	Percent (%)	*n*	Percent (%)	
**In-Hospital Mortality**	92	10.09%	23	15.13%	69	9.08%	0.024
Crude OR (95% CI)	--		1.786 (1.074, 2.967)		1.0		0.025
Adjusted OR (95% CI)	--		1.816 (1.079, 3.056)		1.0		0.025
**30-Day Mortality**	41	4.50%	9	5.92%	32	4.21%	0.353
Crude OR (95% CI)	--		1.432 (0.669, 3.064)		1.0		0.355
Adjusted OR (95% CI)	--		1.431 (0.651, 3.146)		1.0		0.373
**In-Hospital Complications**	18	1.97%	4	2.63%	14	1.84%	0.523
Crude OR (95% CI)	--		1.440 (0.468, 4.436)		1.0		0.525
Adjusted OR (95% CI)	--		1.446 (0.464, 4.501)		1.0		0.525
**Rate of Routine Discharge**	766	83.99%	121	79.61%	645	84.87%	0.106
Crude OR (95% CI)	--		0.696 (0.447, 1.082)		1.0		0.108
Adjusted OR (95% CI)	--		0.682 (0.430, 1.083)		1.0		0.104
**One-Year Recurrence**	199	21.82%	23	15.13%	176	23.16%	0.029
Crude OR (95% CI)	--		0.592 (0.368, 0.951)		1.0		0.030
Adjusted OR (95% CI)	--		0.583 (0.360, 0.945)		1.0		0.029
**Length of Hospital Stay (Mean ± SE)**	--		18.26 ± 1.16		17.18 ± 0.15		0.894
**Hospital Costs (Mean ± SE) ^b^**	--		3914.52 ± 329.89		4109.36 ± 53.06		0.048

Notes: The OR was calculated with a binomial logistic regression. Adjustments were made for the patient’s sex, age stratum, CCI score, hospital level, and cause of the procedure. ^a^ p values for the length of hospital stay and the hospital cost were analyzed with independent *t*-tests, and other variables were analyzed with the chi-square test. ^b^ Costs are expressed in U.S. dollars (USD). In 2007, one USD dollar was equivalent to approximately 32.64 Taiwan dollars.
